# Testicular ischemia associated with IgA vasculitis in a child: a case report and literature review

**DOI:** 10.3389/fped.2023.1219878

**Published:** 2023-08-11

**Authors:** Shuya Zhang, Qingwen Wang, Ziwei Li, Qingyin Guo

**Affiliations:** ^1^ The First Clinical Medical College, Henan University of Chinese Medicine, Zhengzhou, China; ^2^ Department of Pediatrics, The First Affiliated Hospital of Henan University of Chinese Medicine, Zhengzhou, China

**Keywords:** IgA vasculitis, Henoch–Schönlein purpura, children, epididymo-orchitis, testicular ischemia, testicular necrosis, case report, literature review

## Abstract

Testicular necrosis is a rare and severe complication of immunoglobulin A (IgA) vasculitis (IgAV). Herein, We report a case of a 10-year-old boy who was admitted to the hospital due to skin purpura and intermittent abdominal pain for 10 days and bilateral testicular pain for 2 days. Scrotal ultrasonography indicated right testicle ischemia, right epididymo-orchitis, and bilateral hydrocele of the testis. Scrotal surgical exploration revealed significant swelling and darkening of the right testicle. Conservative treatment led to improvement in his condition, and he was discharged. During 3 months of follow-up, there was no recurrence of skin purpura or pain, and the urine tests were normal. Color ultrasound indicated only partial blood flow signal to the right testicle tissue, which was slightly smaller than the left testicle. This case highlights the need for continuous attention from clinicians to the signs and symptoms of the reproductive system during the diagnosis and treatment of IgAV. Continuous monitoring with ultrasound can aid in early detection, diagnosis, and treatment of reproductive system lesions of IgA vasculitis.

## 1. Introduction

Immunoglobulin A vasculitis (IgAV), also known as Henoch–Schönlein purpura, is a systemic immune complex-mediated leukocytoclastic small vessel vasculitis frequently reported among children. Its primary clinical manifestations involve non-thrombocytopenic purpura, abdominal pain, joint swelling and pain, and renal disorders (1). The incidence of IgAV is reported to range from 10 to 20 cases per 100,000 children (2). In general, children with IgAV have a good prognosis, but serious complications such as myocarditis and disorders of the nervous system (such as intracranial hemorrhage), respiratory system (such as pulmonary hemorrhage), and reproductive system (such as scrotal edema, orchitis, testicular torsion) may occur owing to individual differences (3–6). Genital involvement in IgAV is infrequent, and testicular necrosis is extremely rare. To the best of our knowledge, only four cases of testicular necrosis have been reported worldwide (7–10). Recently, a boy with IgAV experiencing abdominal pain and testicular ischemia was treated in our hospital. The abdominal pain was relieved by treatment with glucocorticoid and immunoglobulin, but the partial absence of blood flow in the testicle persisted. In addition, we reviewed previously reported cases of IgAV with testicular ischemic necrosis.

## 2. Case report

The patient, a 10-year-old boy with bilateral testes measuring less than 2.5 cm in long axis and less than 4 mL in volume and no pubic hair, was classified as stage 1 according to the Tanner Stages (11). He was admitted to the Department of Pediatrics of our hospital and presented with a history of skin purpura accompanied by intermittent abdominal pain that persisted for 10 days without relief after treatment. He reported bilateral testicular pain that lasted for 2 days.

**Figure 1:**
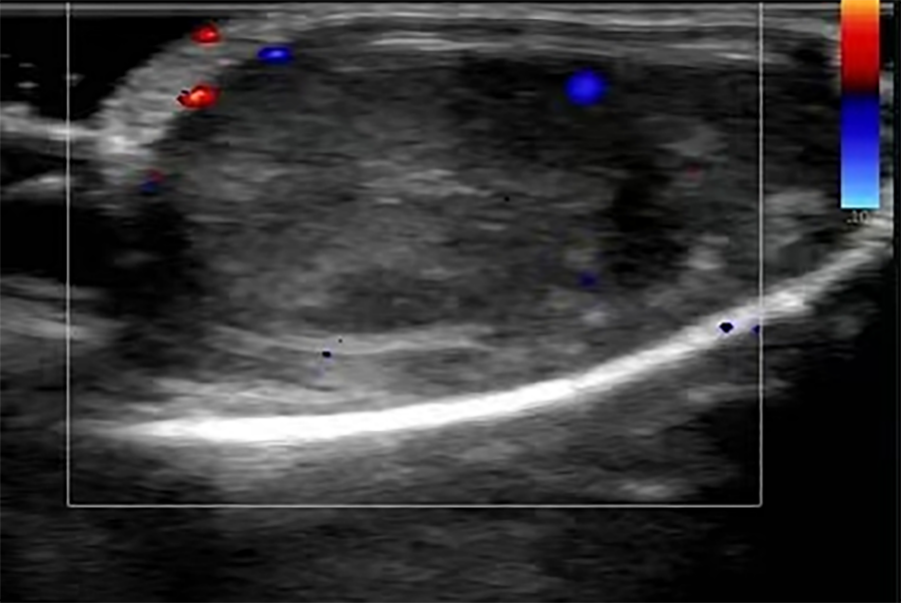
Ultrasound image of the right testis on hospital admission. The color Doppler ultrasound image revealed the uneven echo of the right testis, which showed no significant blood flow signal.

The child developed several skin purpuric lesions on both lower limbs without inducement, accompanied by abdominal pain and knee joint pain 10 days prior to hospital admission. He had normal blood and urine test results and was diagnosed with Henoch–Schönlein purpura. The patient was treated with methylprednisolone 40 mg intravenous drip for 7 days; however, he was discharged despite no relief from abdominal pain . On day 8, he developed bilateral testicular pain that was more severe on the right side. He was transferred to our hospital for treatment on day 10. Physical examination revealed millet-like purpura in bilateral auriculae and both lower limbs, slight abdominal tenderness, and obvious bilateral testicular tenderness. Auxiliary examination showed increased white blood cell (14.3 × 109/L) and neutrophil (10.45 × 109/L) counts. Routine urine and 24-h urine protein results were normal. The patient’s IgA level had increased to 3.15 g/L, but all other laboratory tests were within the normal range. Scrotal color Doppler ultrasound showed that the left testicle was 15 mm × 13 mm × 9 mm in size, exhibiting uniform echo and normal blood flow signal. The right testicle was 16 mm × 10 mm × 8 mm in size, displaying uneven echo and no obvious blood flow signal (Figure 1). The blood flow signal to the right epididymis had increased, along with the presence of a bilateral testicular hydrocele. Combined with clinical practice, testicular torsion was not excluded, and further examination was recommended.

**Figure 2:**
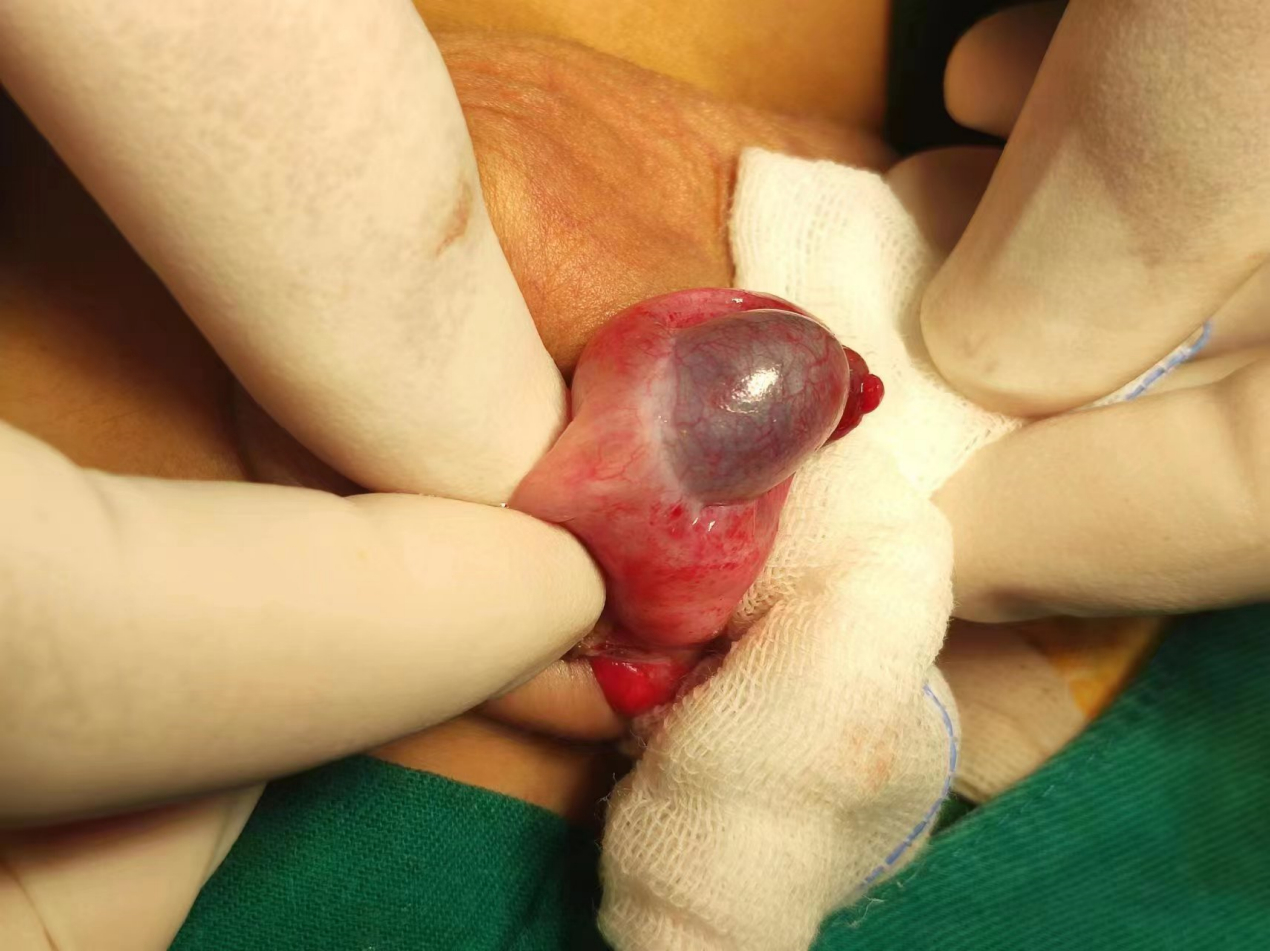
Obvious enlargement and darkening of the right testis due to testicle ischemia owing to vascular occlusion and poor blood supply.

On the day of admission, surgical exploration of testicular torsion was performed under general anesthesia. The surgery revealed a significantly swollen, dark right testicle and no obvious torsion. Testicular ischemia was thought to be caused by vascular occlusion and poor blood supply. Therefore, a warm saline gauze was applied for hot compression. Given the absence of any improvement after half an hour, a lidocaine blockade test was performed. There was no improvement after 20 min of observation (Figure 2). Intraoperative ultrasonic exploration reaffirmed that the echo of the right testicle was slightly uneven and there was no significant blood flow signal. The disease condition was communicated to the family, and it was advised to remove the right testicle. As the family refused this suggestion, the testicle was fixed in the fleshy membrane of the scrotum in its natural position and a rubber drainage strip was placed inside the incision. During the surgery, a grayish-white soft mass of approximately 0.3 cm × 0.3 cm size was detected within the tunica vaginalis of the testis. Subsequent pathological examination under an objective microscope revealed that the lump was a fibrous tissue nodule with a large amount of inflammatory exudation at the center, which contained a small number of fungal spores.

**Figure 3:**
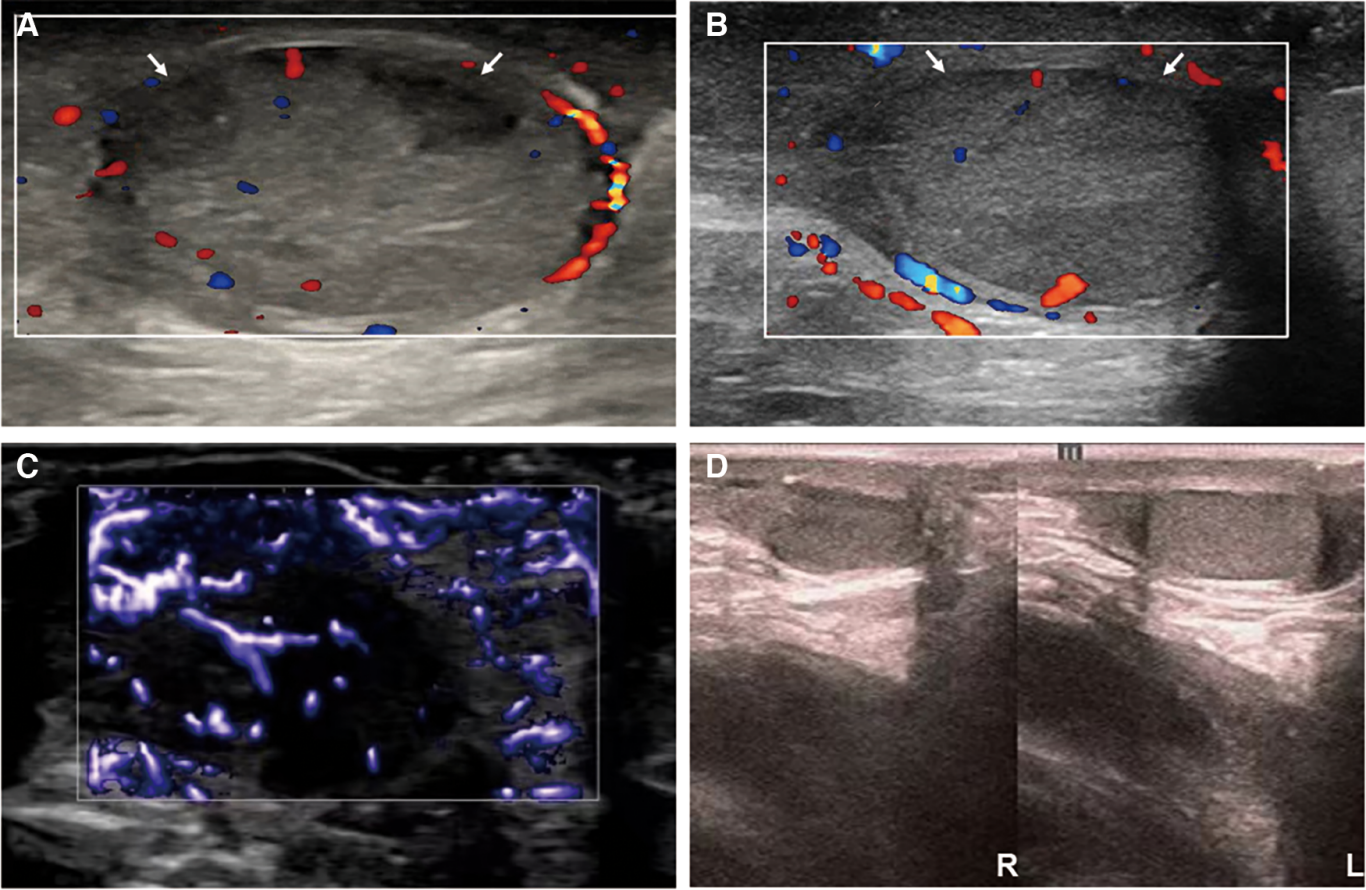
Ultrasound images of the right testicle during treatment and follow-up. From postoperative day 3 (**A**) to postoperative week 2 (**B**), the ultrasound showed two patchy hypoechoic areas (arrow) in the right testicle, which gradually narrowed and almost returned to normalcy. Two weeks after surgery, testicular ultrasound microvascular imaging (**C**) showed a short lines-like blood flow signal in the right testicular tissue. The ultrasound follow-up conducted 3 months after surgery showed that the right testicle was slightly smaller than the left testicle (**D**).

The right scrotum was slightly swollen after surgery, but the left side was normal. The patient was intravenously administered with ceftriaxone sodium, levornidazole, low-molecular-weight heparin, 5 mg/(kg·d) methylprednisolone, and 10 g of human immunoglobulin daily for 2 days. After treatment, the skin purpura gradually subsided, the abdominal pain reduced, and the swelling of the right scrotum was alleviated. Three days after surgery, a color Doppler ultrasound revealed the presence of patchy hypoechoic regions in the right testicle where no significant blood flow signal was detected. The blood flow signals were observed in the surrounding testicular tissue of hypoechogenicity (Figure 3A). Edema of the right scrotal wall and thickening of the right spermatic cord were evident. Ten days after surgery, a color Doppler ultrasound showed areas of patchy hypoechogenicity (the range was smaller than before) in the right testicle, slightly sparse internal blood flow signal, and visible blood flow signal in the surrounding testicular tissue with low echoes along with right scrotal wall edema and right spermatic cord thickening. A color Doppler ultrasound conducted 2 weeks after surgery showed that the internal echo of the right testicle was normal without any abnormal echo areas and the blood flow signal was better than that observed before (Figure 3B). Testicular ultrasound microvascular imaging showed a short-line blood flow signal in the right testicular tissue (Figure 3C).

The patient has been diligently followed up for 3 months after surgery. Throughout this monitoring period, neither recurrence of skin purpura nor pain was observed. Furthermore, urinalysis revealed normal results. Notably, the color Doppler ultrasound highlighted a partial absence of the blood flow signal to the right testicular tissue, which was 13 mm × 8 mm × 6 mm in size and approximately 0.44 mL in volume. The left testicle, on the other hand, was 16 mm × 11 mm × 7 mm in size and approximately 0.87 mL in volume. The right testicle was slightly smaller than the left testicle (Figure 3D). Importantly, the patient has not reported experiencing any discomfort or symptoms of the scrotum.

## 3. Discussion

IgAV is the most common vasculitis encountered in children. The pathogenesis of IgAV is thought to involve humoral immunity abnormalities triggered by infection and other factors, which result in excessive production of galactose-deficient IgA1 (Gd-IgA1). Gd-IgA1 binds to specific antibodies and forms immune complexes that get deposited in the walls of small blood vessels and cause autoinflammation and tissue damage. IgAV mainly affects capillaries but may also involve arterioles and venules, which may increase the permeability and brittleness of blood vessels. There is exudation of serous fluid and red blood cells around blood vessels, infiltration of inflammatory cells, and intimal hyperplasia of small blood vessels, consistent with transparent degeneration and necrosis. This phenomenon causes narrowing of the vascular lumen and infarction and may lead to necrosis of small arteritis (12). The deposition of the immune complex stimulates vascular endothelial damage, activates the coagulation system, and thus mediates a hypercoagulable state, which can lead to ischemia, hypoxia, and tissue damage of the reproductive organs. Therefore, IgAV can be associated with acute lesions of the genitals.

The incidence of IgAV combined with genital involvement has been reported to range from 2% to 38% (13). The vast majority of the cases have been reported in men and involved the scrotum, penis, epididymis, testicles, and spermatic cord. Cases involving testicular blood vessels may be complicated with epididymitis or epididymo-orchitis (7, 8, 10), testicular torsion (14), and spermatic vein thrombosis (9, 15), all of which are risk factors for testicular necrosis that can lead to testicular ischemia and necrosis if not timely treated.

This boy had no history of trauma or intense activity; hence, his testicular ischemia was related to IgAV. Color ultrasound and scrotal exploration revealed no signs of spermatic vein thrombosis and testicular torsion. Given the increased blood flow signal in the right epididymis, edema of the right scrotal wall, thickening of the spermatic cord, and bilateral testicular hydrocele, histopathological examination indicated that the mass in the tunica vaginalis was a fibrous tissue nodule with a large amount of inflammatory exudation visible in its center. Testicular ischemia might be caused by the development of epididymo-orchitis. Inflammatory exudation and edema contribute to the thickening of the spermatic cord sheath and formation of a narrow ring at the transition of the spermatic cord sheath and the testicular sheath. This process leads to the compression of the testicular artery within the spermatic cord and, consequently, to testicular ischemia. Epididymitis or epididymo-orchitis in children with IgAV is related to the involvement of epididymal and testicular blood vessels and may also be associated with infection or compromised immunity due to the use of glucocorticoids. The pathogens can cause epididymitis or epididymo-orchitis by reaching the epididymis through the blood, lymphatic vessels, or vas deferens.

A literature review was conducted using the China National Knowledge Network database and PubMed database based on the terms “Henoch-Schönlein purpura” or “IgA vasculitis,” “testicular ischemia,” “testicular infarction,” and “testicular necrosis.” Publications describing cases of IgAV combined with testicular necrosis were selected. Four case reports described the symptoms, examinations, diagnoses, treatments, and outcomes of IgA vasculitis with testicular necrosis (Table 1).

**Table 1:** Published case reports of IgA vasculitis complicated with testicular necrosis.

Article (year)	Age (years)	Symptoms (scrotum)	Examination	Possible cause of testicular necrosis	Treatment and outcome
Fukuda et al. (8)	12	Left scrotum pain, swelling and enlargement	CRP 1.4 mg/dL↑	Epididymo-orchitis	Surgical examination: Swelling, darkening, bleeding and necrosis of the left testicle
			Serum IgM against mycoplasma↑		
			Serum factor XIII↓		
			CDUS: Blood flow was sufficient inside the left testis and epididymis, but was barely undetectable after 5 days		Left orchiectomy
Zhao et al. (7)	8	Left scrotum pain, swelling and skin redness	White blood cell count (19.47 × 109/L)↑	Epididymo-orchitis	Surgical examination: Swelling of the left testicle with black appearance; ischemic necrosis of the left testicle
			CDUS: No blood supply to the left testicle parenchyma, abundant blood flow to the left epididymis; the right testicle showed a partial hyperechoic area		
					Left orchiectomy
					After methylprednisolone treatment, the hyperechoic area of the right testicle gradually subsided
Yaseen et al. (10)	73	Diffuse tenderness in the scrotum	CRP 12.3 mg/dL↑	Epididymitis	After treatment with prednisone, the symptoms were relieved without recurrence
			ESR 100 mm/h↑		
			IgA 900 mg/dL↑		
			Hematuria, proteinuria		
			Skin biopsy: Positive IgA, IgM, C3		
			CDUS: A large hypoechoic area in the right testicle with absent blood flow, concerning for infarction		
Toushan et al. (9)	51	Scrotal skin purpura, pain, edema	CRP↑	Bilateral spermatic cord vasculitis-associated thrombi formation	Methylprednisolone + dapsone
			IgA 431 mg/dL↑		Bilateral testicular infarction orchiectomy
			CDUS: Bilateral scrotal wall edema, insufficient testicular blood flow without any evidence of torsion		

CDUS, color Doppler ultrasound.

Among three patients with epididymitis or epididymo-orchitis, one presented a large hypoechoic area in the right testicle without blood flow in a scrotal ultrasound examination, which was related to infarction. The symptoms were alleviated following treatment with prednisone (10). The other two cases developed left testicular necrosis and underwent testicular resection (7, 8). Bilateral testicular necrosis occurred in one patient with bilateral spermatic cord thrombosis, which was resolved by orchiectomy (9).

While IgAV is more frequent in children, it is clinically more severe in adults, who often exhibit more severe clinical manifestations and poor prognosis, including severe kidney damage. However, the reasons underlying this difference in clinical presentation related to age of onset are unclear. One hypothesis centers around the combined effects of comorbidities and aging. Potential precipitating factors such as infection are more common in children than in adults, and the susceptibility to upper respiratory tract infections in children may be associated with the increased incidence of IgAV in pediatric patients (16). Conversely, testicular necrosis is infrequent, and there has been limited investigation comparing adults and children in this regard.

Previous findings have recommended early treatment with glucocorticoids in children with IgAV who experience gastrointestinal symptoms, nephritis, arthritis, pulmonary hemorrhage, and acute vasculitis of the reproductive organs (17). IgAV complicated with epididymitis or orchitis is mostly a self-limiting disease and is effectively treated with glucocorticoids and antibiotics (18). The acute phase is often accompanied by a hypercoagulable state characterized by abnormal levels of various coagulation factors and can be managed by the addition of anticoagulants. Diana et al. (15) reported an 8-year-old boy with IgAV who experienced acute pain and swelling of the left scrotum. Exploratory surgery revealed extensive thrombosis of the spermatic vein. The patient was treated with low molecular weight heparin that alleviated thrombosis, as observed in the ultrasound examination.

For male children with IgAV, attention should be paid to conducting genital examinations combined with dynamic observation of the color Doppler ultrasound. In the case of testicular torsion, it is necessary to obtain time for surgical reduction as soon as possible and strive to complete the operation within 6 h of the appearance of symptoms. Kajitani et al. (14) reported a case of a child with testicular torsion who had decreased blood flow in the center of the testis. Testicular torsion reduction fixation was performed in time to avoid any occurrence of testicular necrosis. Testicular necrosis is a surgical emergency. The ischemic necrosis of the testicle is accompanied by the destruction of the blood–testis barrier, which may lead to the production of antisperm antibodies and the triggering of autoimmune reactions, and even involve the healthy testicle. The consequences can result in changes in sperm quality or immune infertility. Therefore, early surgical removal of the affected testicles is warranted (19).

In this particular case, there was no obvious blood flow signal in the testis in the acute stage neither was any improvement in the condition after half an hour of hot compression with warm saline gauze. The parents refused orchiectomy. After medical treatment, the symptoms were relieved and partial restoration of testicular blood flow was observed. Future studies are needed to determine the optimal timing and indication for testicular resection in such cases. In addition, follow-up is necessary to evaluate the long-term prognosis of children with IgAV involving the reproductive system and its potential impact on future fertility.

In conclusion, IgAV can affect not only the skin, gastrointestinal tract, joints, and kidneys but also the reproductive system. Nevertheless, the presence of testicular involvement has been largely underestimated in a large series of children with IgA vasculitis (20). Genital involvement in children with IgAV may lead to testicular necrosis if not diagnosed and treated in time, suggesting that clinicians should pay more attention to the occurrence of such rare complications in conjunction with ultrasonography during the diagnosis and treatment of IgAV. It is important to consider the potential risk of this complication in boys with IgA vasculitis. Consequently, a thorough clinical evaluation should be performed to exclude testicular ischemia in these patients.

## Data Availability

The original contributions presented in the study are included in the article/Supplementary Material, further inquiries can be directed to the corresponding author.
